# Climate change adaptation must not replicate lockdown scenarios

**DOI:** 10.1177/17579139241231130

**Published:** 2024-08-06

**Authors:** Philip Weinstein, Peng Bi, Jessica Stanhope

**Affiliations:** School of Public Health, The University of Adelaide, Adelaide, SA, Australia; Environment Institute, The University of Adelaide, Adelaide, SA, Australia; School of Public Health, The University of Adelaide, Adelaide, SA, Australia; Environment Institute, The University of Adelaide, Adelaide, SA, Australia; School of Allied Health Science and Practice, The University of Adelaide, Adelaide, SA, Australia; Environment Institute, The University of Adelaide, North Terrace Campus, Adelaide, SA 5005, Australia

This paper highlights concerns related to climate change adaptation strategies, including staying indoors and using air conditioning, as these strategies will lead to similar issues as faced with the COVID-19 lockdowns, including social isolation, reduced physical activity and reduced exposure to green space. These are critical concerns in light of climate change.

There is now an overwhelming body of evidence that climate change is and will continue to adversely affect health, including heatstroke, adverse pregnancy outcomes, worsened kidney function, adverse mental health effects, reduced labour productivity, and threatened livelihoods – and through its environmental impacts, poses a threat ‘to our very survival and to that of the ecosystem upon which we depend’.^1^ A broad range of responses is urgently required not only to arrest climate change at its source (fossil fuels) but also to minimise the adverse health impacts of an already locked-in climate change momentum.

One such adaptation is improved housing, to insulate us from the effects of increased intensity and duration of heat waves – and the wish to do so has already resulted in a huge increase in the number of houses with insulation, ceiling fans, and air conditioning.^2,3^ Air conditioning can be seen as ‘a maladaptive response that worsens the energy crisis and further increases urban heat, air pollution, and greenhouse gas emissions’,^3^ with improved insulation and ceiling fans providing better options for reducing fossil fuel use. While such climate change adaptive measures may reduce our extreme heat exposure, relying on such housing improvements comes with its own health risks – namely being a virtual prisoner in your own home to avoid extreme heat exposure, very much like during the COVID-19 lockdowns.

The COVID-19 experience provided an insight into what our future may look like if we rely upon the protection of our houses as a climate change adaptation strategy. The benefits of physical isolation and quarantine for avoiding infection go without saying, but there are also subtle negative outcomes from staying indoors, related to physical isolation, fewer opportunities for exercise, and isolation from nature.^4^

Social isolation is a likely consequence of physical isolation and has been associated with increased all-cause mortality, cardiovascular disease, and poorer mental health.^5^ Indeed, Butterworth et al.^6^ found that lockdowns to control COVID-19 resulted in poorer mental health. Similarly, staying indoors may reduce opportunities for free and accessible exercise and reduced exposure to green spaces. The negative consequences of reducing exercise are clear, and those who reduced their exercise during the COVID-19 pandemic experienced poorer mental and physical health.^7^ These adverse health outcomes may increase if we rely upon staying indoors to avoid heat waves.

**Figure:**
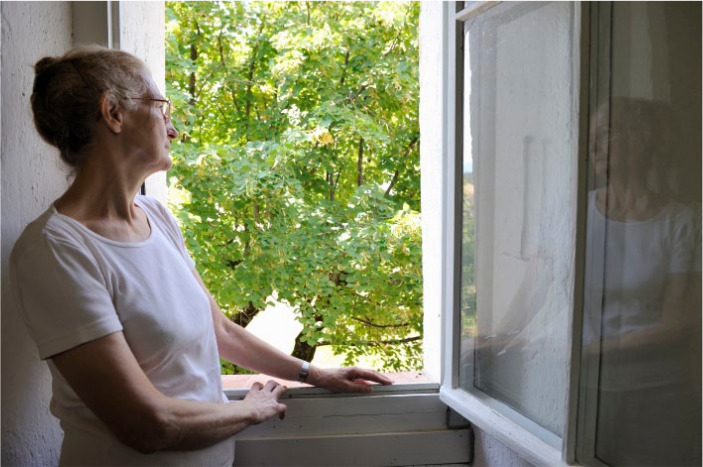


Furthermore, we are increasingly understanding the importance of green space exposure in determining mental and physical health, including all-cause mortality.^8^ There is a range of elements to green space exposure that may explain these associations. Many of these elements, such as reductions in air, noise and light pollution, as well as reduced heat-island effects,^8^ are not dependent on an individual spending time in green spaces, but rather allow public health benefits through the strategic placement of green spaces in proximity to residential spaces. However, other elements of green space do require direct access to optimise health benefits therefrom. For example, nature-based virtual reality does not have the same health benefits as actual green space exposure.^9^ Similarly, exposure to environmental microbiota may influence the human microbiota and therefore human health,^10,11^ and direct exposure to soil and vegetation is likely to have a stronger effect than does aerobiome exposure. Even though the biodiverse aerobiomes from green spaces may influence health outcomes without requiring direct green space contact,^10,11^ to benefit people would still need to be in close proximity to a biodiverse green space (within 400 m),^12^ and have their windows open – which is unlikely if in an air conditioned home during a heat wave.

One could argue that heat waves do not last as long as some COVID-19 lockdowns, however, longer and more frequent heat waves are predicted to affect our health in the future under the majority of climate change scenarios. We highlight the importance of including consideration of the adverse health effects of weather-induced isolation when creating domestic refugia against climate change. If we do not account for the potential adverse health effects of spending more time indoors, we might unwittingly exacerbate the situation with recommendations that do not appropriately balance indoor and outdoor exposures. Retreating indoors may be one weapon in our climate change adaptation armamentarium, but we must not replicate the adverse health effects of lockdowns as revealed by the COVID-19 pandemic.
